# Health, Health Care, and Systems Science: Emerging Paradigm

**DOI:** 10.7759/cureus.1030

**Published:** 2017-02-15

**Authors:** Ivo Janecka

**Affiliations:** 1 Research and Education, Foundation for Systems

**Keywords:** health, health care, systems science

## Abstract

Health is a continuum of an optimized state of a biologic system, an outcome of positive relationships with the self and others. A healthy system follows the principles of systems science derived from observations of nature, highlighting the character of relationships as the key determinant.

Relationships evolve from our decisions, which are consequential to the function of our own biologic system on all levels, including the genome, where epigenetics impact our morphology. In healthy systems, decisions emanate from the reciprocal collaboration of hippocampal memory and the executive prefrontal cortex.

We can decide to change relationships through choices. What is selected, however, only represents the cognitive interpretation of our limited sensory perception; it strongly reflects inherent biases toward either optimizing state, making a biologic system healthy, or not. Health or its absence is then the outcome; there is no inconsequential choice.

Public health effort should not focus on punitive steps (e.g. taxation of unhealthy products or behaviors) in order to achieve a higher level of public’s health. It should teach people the process of making healthy decisions; otherwise, people will just migrate/shift from one unhealthy product/behavior to another, and well-intended punitive steps will not make much difference.

Physical activity, accompanied by nutrition and stress management, have the greatest impact on fashioning health and simultaneously are the most cost-effective measures. Moderate-to-vigorous exercise not only improves aerobic fitness but also positively influences cognition, including memory and senses. Collective, rational societal decisions can then be anticipated.

Health care is a business system principally governed by self-maximizing decisions of its components; uneven and contradictory outcomes are the consequences within such a non-optimized system.

Health is not health care. We are biologic systems subject to the laws of biology in spite of our incongruous decisions that are detrimental to health. A biologic system/a human body originates from structural, deterministic genes as well as shared epigenetic memory of our ancestors affecting our bodily function and structure. The political governing systems’ vertical hierarchy has control over money and laws, neither of which materially affect individual lifestyle/behavioral choices toward health. Improved health comes from focusing on enhancing the biologic age and not the chronologic one, which simply represents a linear time from a birth certificate to a death certificate and is applicable only in its extremes. “Age-related diseases” are simply reflections of a given culture. Biologic age, reflecting the actual state of health, could be used in all health-related assessments including health-life insurance premiums, licensing of job categories, etc., all with a broader and healthy societal impact.

## Introduction and background

Whatever exists in the universe or on planet Earth is a system that is in relationship with the self and other systems, creating patterns and following cycles. Both patterns and cycles reflect a degree of self-similarity and self-affinity characteristic of healthy fractal forms in nature. These relationships are either rule-based (per physics, chemistry, genetics, etc.) that only change over long periods of time or are cognition/decision-based, which can change within a very short time.

Life has been sustained on planet Earth for millions of years and without human help. As a consequence, life has an unprecedented historic record of favorably sustaining itself, through adaptation and evolution, growth, and decluttering/recycling of its components. Ex post facto, humans would do best to defer to and respect the wisdom of that experience for the sake of their own survival and for being an ecologic fit. Nature does not need human leadership, it only needs our realization that all systems are interconnected in the never-ending dance of “your output is my input, and my output is somebody else’s input.” Simply put, our decisions affect all of us in some way, for better or for worse.

How we fit into the rhythm of life depends on, to a large extent, how we, individually and collectively, create our choices that guide our behavior; those choices/decisions ultimately generate epigenetic influence, the up/down functional regulation of most of our structural genes, which may or may not be contributing to the sustainable health of given biologic systems.

What is selected as a choice by “free will,” only represents cognitive interpretation of our limited sensory perception, preceded by selected sensory processing; it strongly reflects inherent biases toward either optimizing, making a biologic system healthy, or not (we “see” what we want to “see”). Health or its absence is then the outcome; there is no “free lunch” or an inconsequential choice.

The term, complex adaptive system, summarizes the positive attributes of a healthy system that is capable of producing value, its emergence, through organized complexity. Any disharmony in relationships has the capacity to alter organized into disorganized complexity and the likely consequential absence of value creation, a portrait of an unhealthy system. Complexity has a major impact on the system’s capacity to manage change. The significance of maintaining a state of health of any system is prima facie. A healthy human body is the best known complex adaptive system composed of numerous smaller sub-systems, all the way to cells, microorganisms, viruses, etc. Complex adaptive systems cannot be micromanaged and successfully live; they have to be permitted to engage in a complementary self-organization of components and be also optimized by governing hierarchy in order to achieve rich organized complexity.

The term “health” is in ubiquitous use today, but it belongs to a category of nebulous words with meanings and goals as different as are people. In spite of their dominant presence in the current lexicon, nebulous words don’t represent optimized, interoperable communication among cognition-based biologic systems, e.g. humans, as they often mislead and confuse. Such terms need to be clarified, for example, a discussion pertaining to a state of health, needs to include what actually must be accomplished, defined as measurable levels of comprehensive/global physiologic performance, in order to sustain the healthy mosaic of a larger system, e.g. entire human body, community, society, etc. The word “health,” in this text, means a global/comprehensive fitness encompassing endurance, speed, strength, and flexibility of aerobic performance supported by amalgamation of nutrition, moderate-to-vigorous exercise leading to fitness, and stress management. Such a healthy system is capable of ongoing adaptation and evolution because it generates value as its output/emergence defined/made visible through efficiency, effectiveness, risk management, and proportionate cost.

Absence of illness cannot be equated with health. The first is just a point in time without a diagnosed disease, but the second, health, is a continuum of an optimized biologic system; as an analogy, taken from the business world, a point in time is represented by a balance sheet, a snapshot of an organization at a specified date vs. a statement of operation which shows continuing performance over a period of time.

This study has searched for key determinants of biologic systems' healthy sustainability. It started with a closer look at what a system is, how its relationships impact the formation of system’s complexity and its eventual output, health, as the emergence.

### Research hypotheses 

Is it true that for cognition-dependent systems, not everything that seems logical to be healthy actually is so? Can only healthy/optimized living/biologic systems generate value with the outcome of sustainable health? Is systems science highly capable of differentiating the degree of healthy sustainability among systems? Is the human body the best known complex-adaptive system, and can it serve as a model for judging other biologic systems? Is there a key portion of the neuro-net that directs biologic systems toward optimized/healthy or non-optimized/unhealthy decisions?

## Review

The period from the 1960s to the present has been selected for the study of public domain records in the context of systems science and their possible relevance to healthy sustainability.

In addition to scientific articles, general public domain publications were also selected, especially the ones describing characteristics of a larger societal system. The collected information was grouped by similarities or dissimilarities with systems science principles. For example, for entropy, reports of decline and diminishing functionalities of systems were extracted; for chaos, publications highlighting exponential excess of unmitigated growth without differentiation, were mined. For systems in health territory, descriptions of optimal adaptation and evolution were also examined.

**Figure 1:**
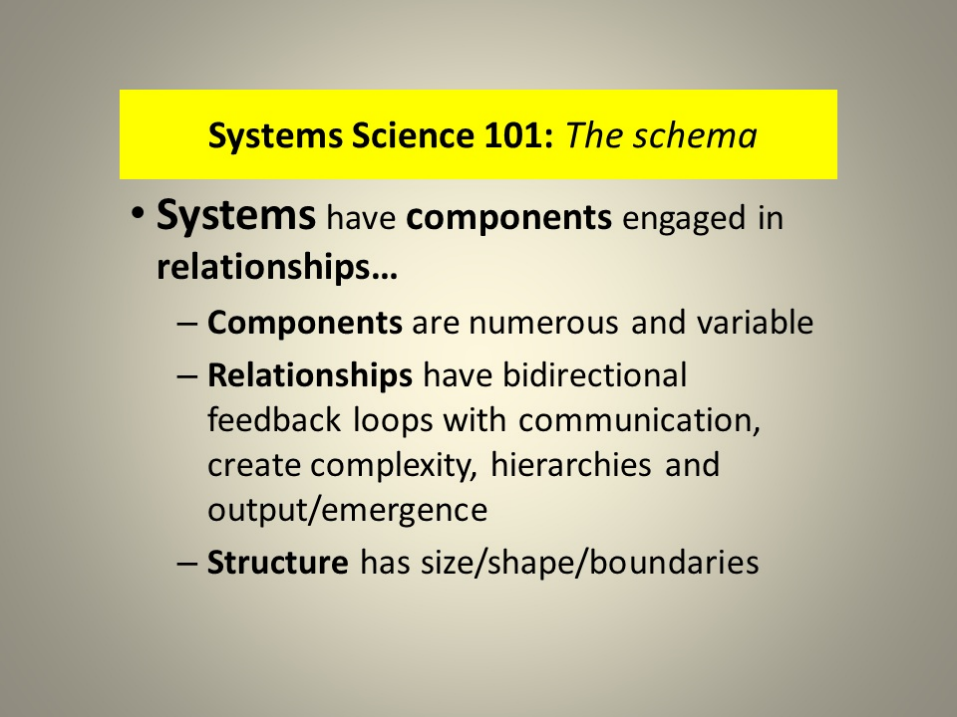
Systems Science Relationship Schema.

**Figure 2:**
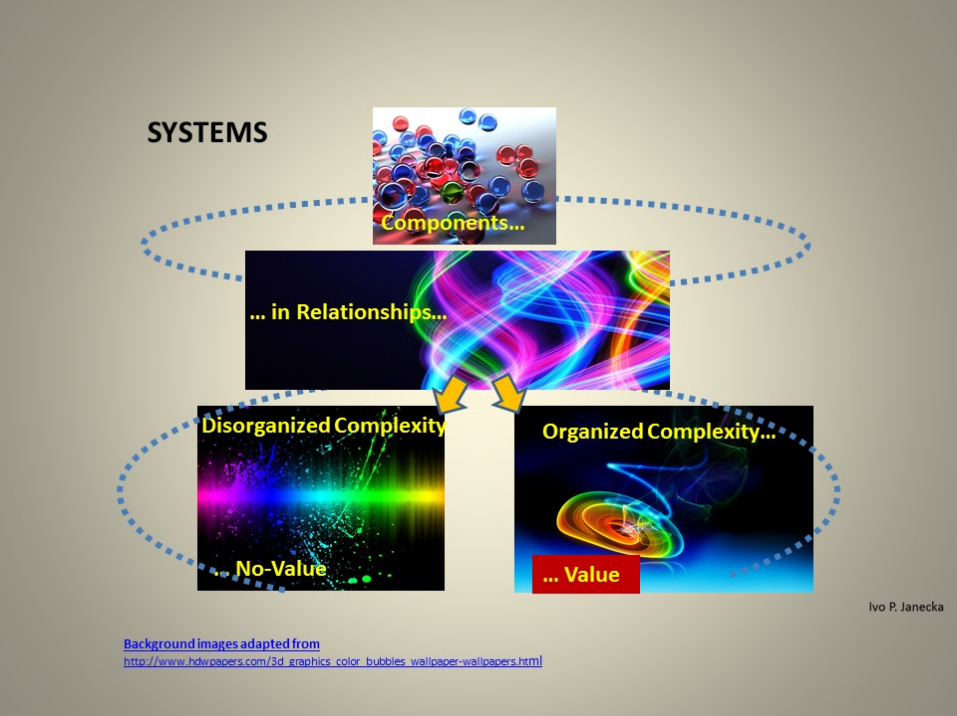
Systems Science Graphic Schema.

**Figure 3:**
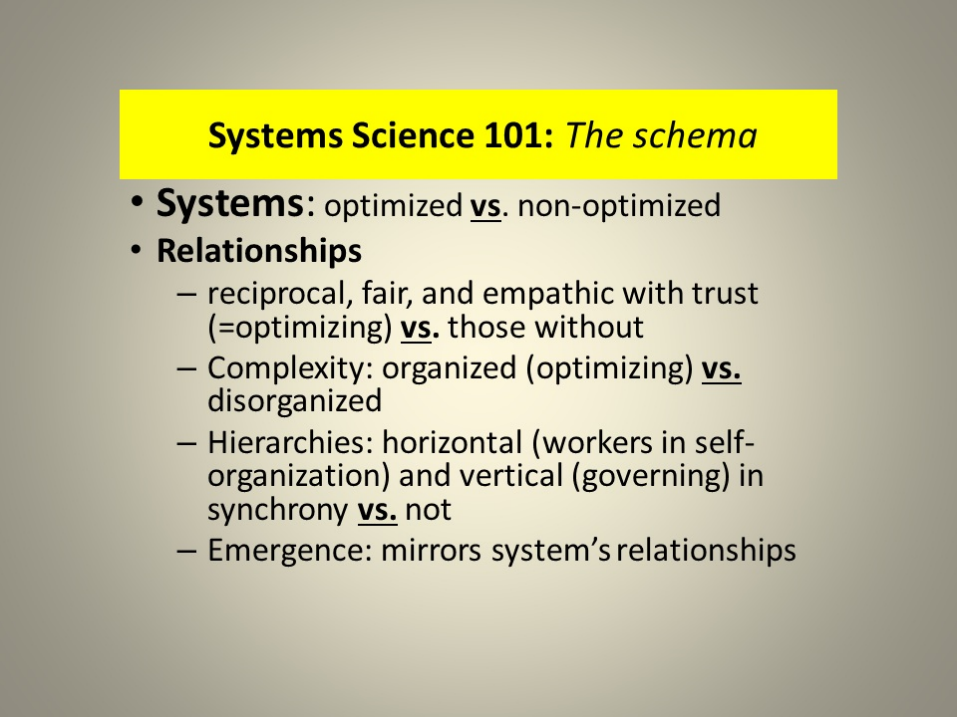
Systems Science Relationship Schema.

Systems Science and the Dynamic Systems Model methodologies were used in this study. They offer a complementary perspective on examining sustainability (Figures 1-3).

Systems science is pertinent to this study because it describes the characteristics of systems. Systems have components/sub-systems, from cells to societies, which should be numerous and variable but with a high degree of shared decision rationality. Systems have relationships with feedback loops which, in healthy systems, express reciprocity, fairness, cognition-dependent empathy/caring, and trust. Relationships create functional complexity which produces the eventual outcome, system’s emergence. When some of the above-listed features are absent from relationships, the gaps serve as warning of potential instability and fragility of such a system. Systems science differentiates between healthy and unhealthy systems, those with the highest capacity to extract meaning from change, and those with the least. The utilization of systems science is valuable for the evaluation of sustainability, the healthy adaptation and evolution with change, in a multidimensional/non-linear/fractal contextuality. Such an approach offers different and likely more robust interpretation of what is happening and how best to respond.

Gradient flow of energy and information, within a function-defined size, shape, and boundary is essential to the survival of any biologic system. It refers to the intake-throughput-output sequence that creates a directional and proportionate cascading balance from intake to output. Energy as well as information intake have to be managed to maintain such a flow through a boundary that is semipermeable; unfiltered intake of energy and/or information overwhelms the throughput, making rational output/decisions difficult and, consequently, creates an unhealthy system.

The system’s boundary needs to be dynamically semipermeable with intake and output thresholds; the system's size should reflect balance between function and structure, one that allows for oscillation with related cycles. Healthy systems demonstrate a system-within-a-system patterns of self-affinity and self-similarity; fractal patterns that such systems generate can be considered a reflection of sustainable adaptation and evolution.

Complexity, arising from optimizing relationships, incorporates processes of self-organization within horizontal hierarchy (“people/components in the trenches”) and a governing vertical hierarchy (which creates rules, laws, their enforcement, institutions, tariffs, etc.). Organized/optimizing/healthy complexity gives rise to collective intelligence of a system that encompasses collective rationality and responsibility based on commonly accepted scientific facts—a potential for creating value/its emergence, is present; such a system is robust enough to express a high degree of resiliency and functional redundancy. Lack of organized complexity, expressed as disorganized complexity, leads to the absence of value creation and cognitive dissonance/stress.

Every well-functioning system, regardless of its size, needs a semipermeable boundary, a filter, for incoming and outgoing data/information as well as energy for meaningful input-output sequence. A system boundary is a reflection of the complexity character of a given system that is enclosed/protected by such periphery but with ongoing intra- and inter-systems balanced relationships, such as blood-brain barrier, intestinal lining, as well as those that are cognition-based, such as boundaries with parents, teachers, society, etc. Those systems that are in the Health Territory will have optimizing/electively transgressible boundaries; the non-optimizing systems have boundaries that are either too rigid or too loose, either severely filtering any input (think of arteriosclerosis depriving tissue of oxygen) or an indiscriminate input that is overwhelming the system (think of cancer invasion or obesity).

Dynamic Systems Model complements Systems Science because it places systems of various complexity states in a multi-dimensional assessment pattern. Such a mosaic allows for a more accurate creation of meaning from any perceived change [1].

**Figure 4:**
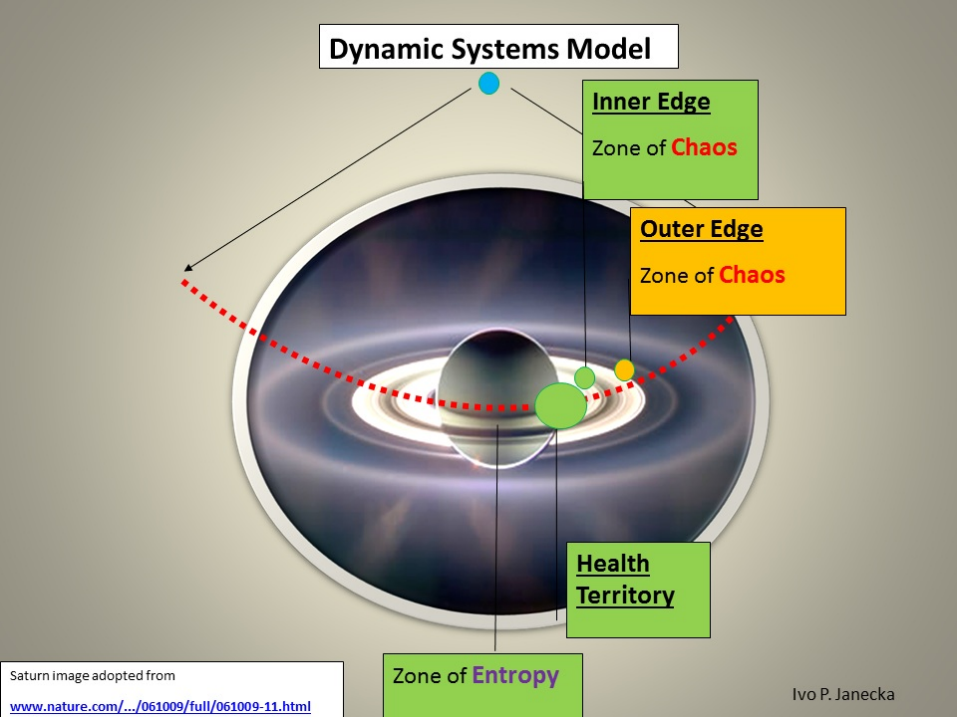
Zones of Dynamic Systems Model.

**Figure 5:**
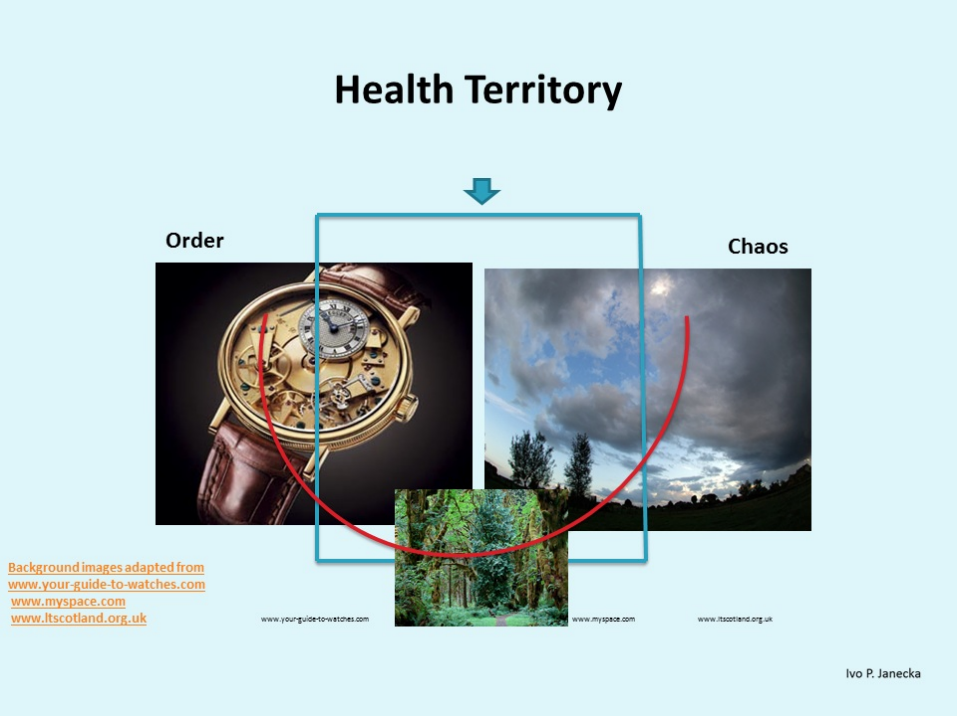
Dynamic Systems Model: Health Territory Visual Schema.

**Figure 6:**
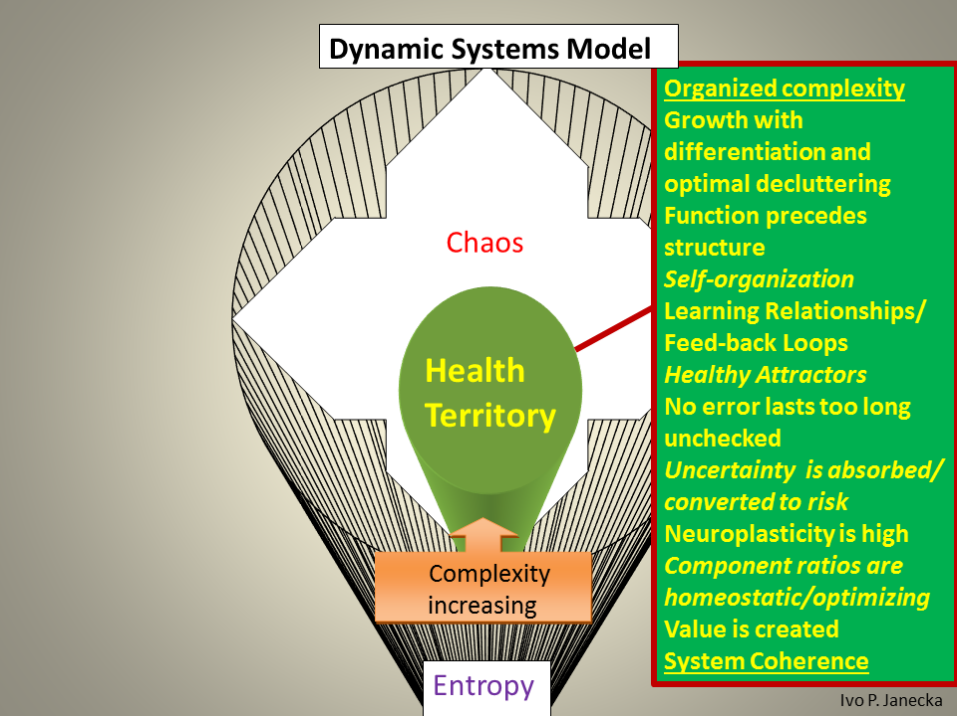
Characteristics Found in Health Territory.

**Figure 7:**
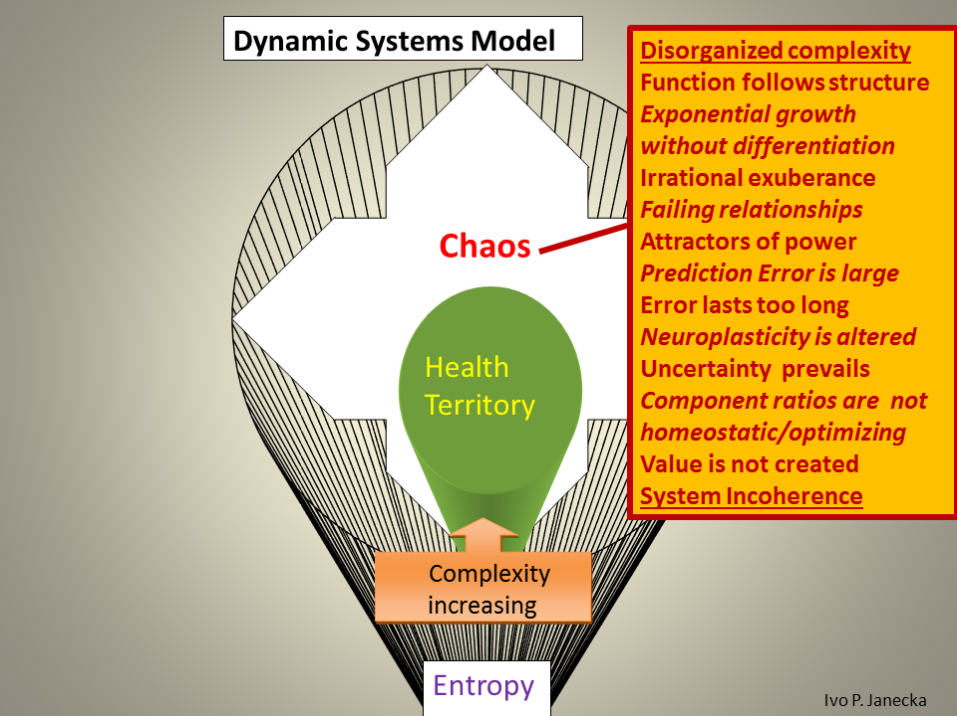
Characteristics Found in Chaos.

**Figure 8:**
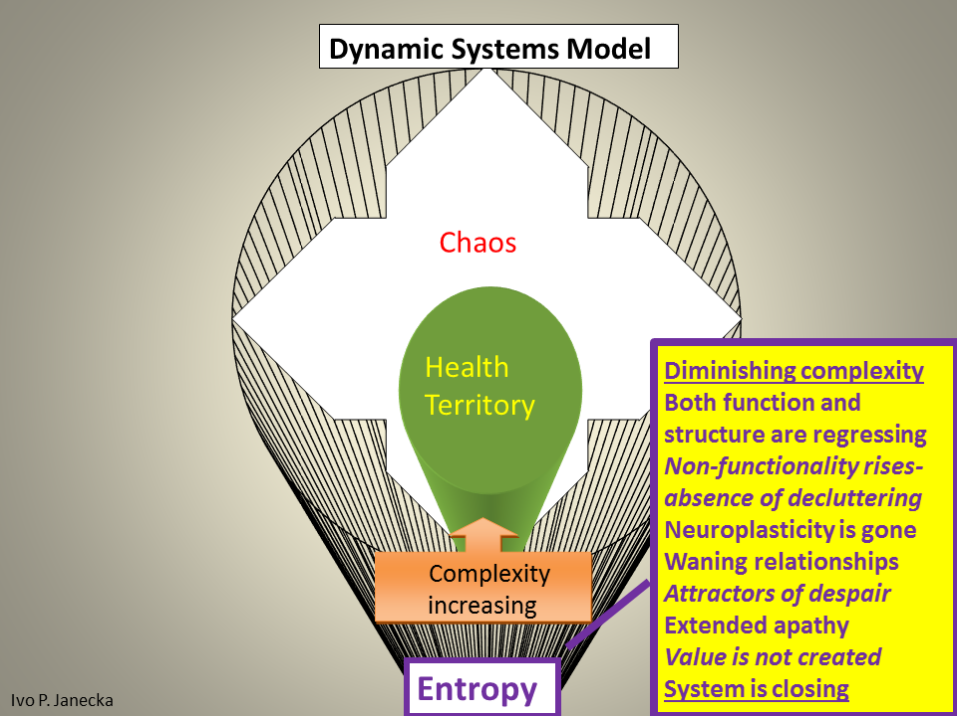
Characteristics Found in Entropy.

**Figure 9:**
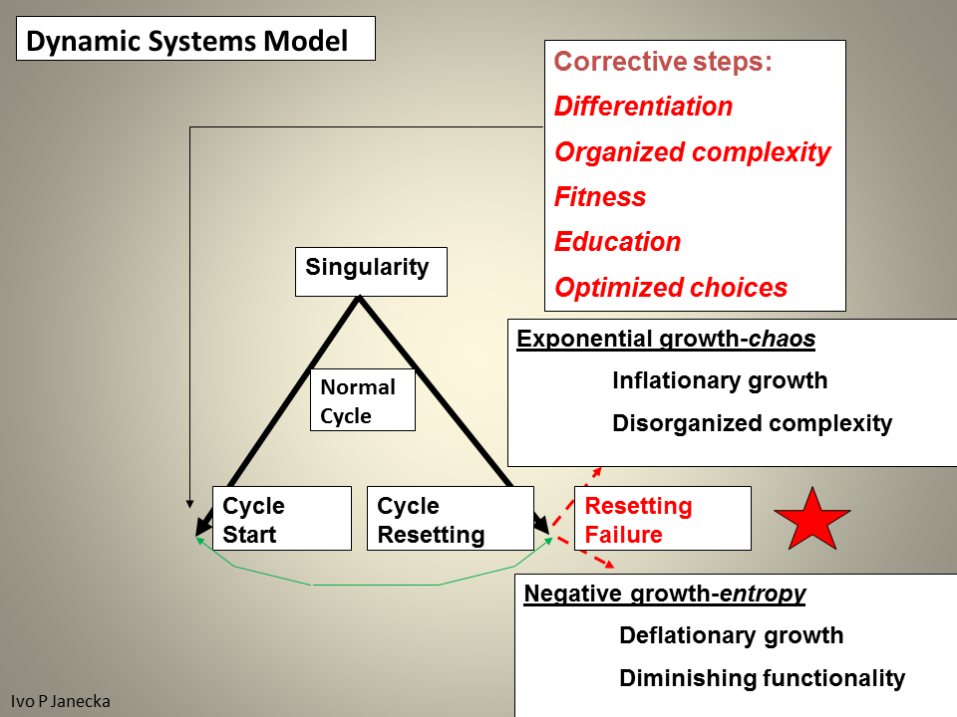
End-state Features of Chaos and Entropy as Well as Feasible Corrective Steps.

The model defines three zones based on their functionality. The first zone—the Health Territory—indicates optimal functioning of a healthy system. This zone dynamically spans the boundaries of two neighboring zones, the outer core of Entropy and the inner edge of Chaos. Extremes, the outliers in this model, include the outer edge of chaos and the inner core of entropy. Each classification within this model reflects, on the one hand, the degree of the system’s capacity for organized complexity—adaptation and evolution—as well as the ability to produce positive emergence/value, on the other hand, it also reveals the high likelihood of random events or system entropic end-phase. Health Territory produces evolutionary changes, chaos generates revolutionary changes, entropy generates devolutionary changes (Figures 4-9).

 

When a system is in Health Territory, random events are rare; most randomness is absorbed/dealt with proactively by a system that is fit. Because randomness is infrequent, it is possible to anticipate it only as an unlikely event; the interpretation of sensory input has the highest probability of being meaningful.

When a system is in chaos, randomness is the norm and the time frame for resolution is short. Events in chaos may lead to anarchy (summation of randomness and asynchronous cycles); abnormal DNA methylation can be observed and measured, demonstrating rapid cellular aging.

The state of a system, either healthy or unhealthy, in Health Territory or not, can be determined with a great degree of accuracy by also looking at the type of past and present relationships as well as the historic and current outputs that have been generated and observed; this insight offers advanced knowledge of what can be anticipated from any new incoming sensory input prior to the time of sensory perception.

**Figure 10:**
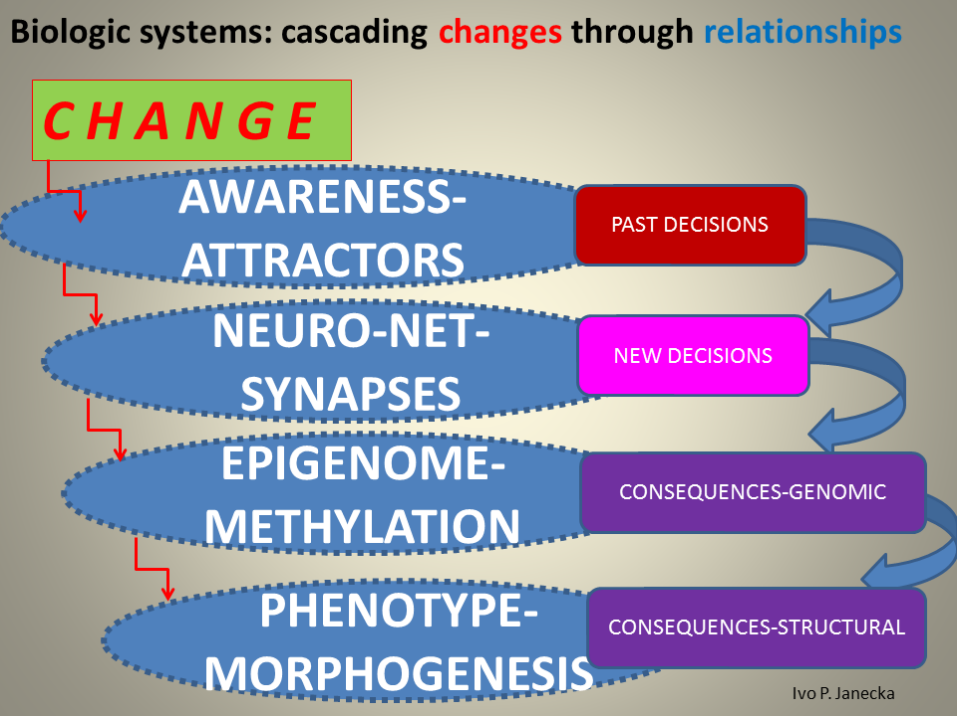
Schema of a Propagating Change Through Levels of Biologic Systems.

**Figure 11:**
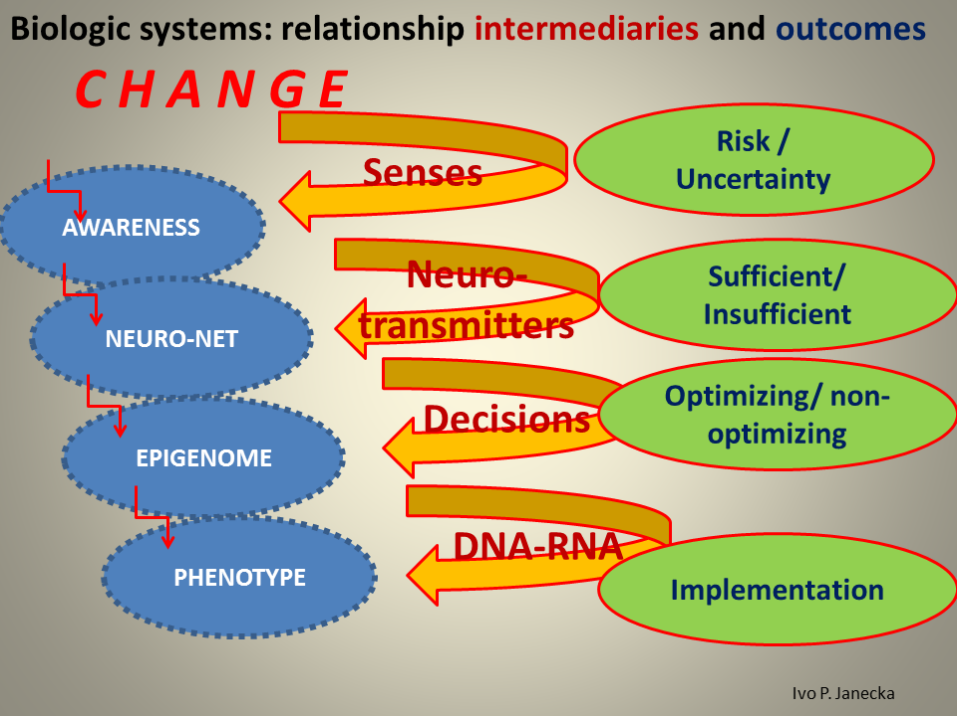
Options That May Activate Systems' Paths.

The evolutionary sustainability of biologic systems, us included, depends on how we all fit into the fundamental rhythm of life, which is intimately tied to our decisions; those decisions are based jointly on our memory and the prefrontal executive cortex, both created by past sensory processing and perception. It is a rolling forward engagement of input-processing-decisions sequence where past memories affect current decision-making and that, in turn, reconfigures with updates, our existing memories (Figures 10-11).

 

We are our memory, which constructs our reality, our narrative. Existing patterns of memory are recalled with any new event acquisition for a probabilistic pattern comparison. Such a fundamental memory-acquisition-recall sequence, however, immediately highlights the critical importance of memory formation by a given biologic system and is raising the following question, pertinent to both a system generating change as well as one that is observing/assessing it: was an optimized hippocampus-prefrontal cortex relationship engaged? A healthy biologic system preferentially selects three-dimensional (3-D) sensory input, known to form patterns of 3-D memory neuro-network within the hippocampus. In contradistinction, an artificial two-dimensional (2-D) sensory input, for example from flat screens of TV, phones, simulators, etc., leads to a loss of 3-D memory. Any subsequent recall of such inferior memory pattern from the hippocampus then provides only a mediocre/unstable substrate for any probabilistic pattern comparison of future sensory input. Also, if the preferential pathway for sensory processing is the reward center route, then the prefrontal cortex, the executive decision center, is significantly handicapped to make healthy/optimized decisions [2-5].

The 3-D or 2-D memory construction in the hippocampus has significant consequences for managing life events. For example, if the primary training of pilots, surgeons, etc. is in simulators, it creates deceptive learning and skill mal-development as none of the trainees will always be flying or operating on a simulator. The autopilot, for example, has programmed limits on its range of corrective maneuvers and when it disconnects, a live pilot has to take over, but then he is not in a simulator but is facing a 3-D life emergency which likely is outside of the programmed simulator teaching. The gap between 2-D training and 3-D life situation has already resulted in plane crashes; training in simulators should be delegated to a supporting role which must be far surpassed by real-life experiences.

Virtual reality/simulation, the future existential hope of many, provides vastly inferior input to the neuro-net which needs to create its own space-mapping framework; a “flat screen” input likely leads to a “flat hippocampus.”

The enormous amount of “screen time” that the current generational cohorts are engaged in, has potentially a significant negative impact on our individual and collective neuro-net memory, as the most recent laboratory recordings from the hippocampus indicate. “Screen time” provides infinitely inferior sensory input into the neuro-net which appears to lack recallable memory patterns and represents disorganization. A screen time often delegates learning/memory to some hard drive or a cloud server and not the hippocampus. Without recallable integrated patterns of core knowledge within the neuro-net, new brilliant discoveries will likely be harder to formulate. Simulation/screen time, regardless how seductive, is not life. Teaching and learning must primarily engage life [6-9].

The pattern of activity in a brain region involved in spatial learning in the virtual world is completely different than in the real world when the hippocampal neurons become selectively active, providing a "cognitive map" of the environment. In the virtual world, in a laboratory, rats' hippocampal neurons seemed to fire completely randomly, as if the neurons had no idea where the rat was—even though the rats seemed to behave perfectly normally in the real and virtual world. The original real world 'map,' recorded with electrodes, disappeared completely. The neuron activity was a random function of the rat's position in the virtual world. The hippocampal neurons were highly active in the real-world environment but more than half of those neurons shut down in the virtual space. The virtual world used in the study was very similar to the virtual reality environments used by humans, and neurons in a rat's brain would be very hard to distinguish from neurons in the human brain. Complex rhythms of neurons are crucial for learning and memory; they are using two entirely different languages: one is based on rhythm [movement], the other is based on intensity. Every neuron in the hippocampus speaks the two languages simultaneously and those neurons involved in memory have to be perfectly synchronized [3].

Devout video gamers may develop more efficient visual attention, but it is likely that the path from senses to cognition mostly bypasses the hippocampus and travels instead through the reward center of the caudate nucleus, etc. as, indeed, videogames are driven by “rewards” [9].

It is estimated that the average young [video gamers] will have spent some 10,000 hours gaming by the time they are 21. They may exhibit more efficient visual attention but more likely use navigation strategies that rely on the brain's reward system (caudate nucleus) and not on the brain's spatial memory system of the hippocampus. People who use caudate nucleus-dependent navigation strategies have decreased grey matter and lower functional brain activity in the hippocampus and may have reduced hippocampal integrity, which is associated with an increased risk of neurological disorders such as Alzheimer's disease [8].

The universe, our planet Earth, and the ecology including the biological systems are all open systems requiring ongoing forward-biased gradient flow of energy and information through phases of intake—metabolism/throughput and output. A proportionate decluttering/ recycling must be present in lockstep; any attempt to maintain status quo results in stagnation.

All biologic entities are considered systems; while some are optimized, others are not. Thus, not everything that seems logical to sustain should be because only a healthy/optimized biologic system can generate value with outcome of sustainability; systems science is highly relevant to differentiate value-based from value-less sustainability; the human body can serve as a model.

Patterns and cycles are indigenous to healthy systems. One of the key cycles, the circadian rhythm—the light and darkness oscillation—is common to all living systems, creating a cadence of activities with different focus and importance. For example, the presence of light serves as a background for the majority of our sensory input while darkness permits pruning and categorization of received data. Cognition, and thus decisions, are strongly affected by the degree of balance of these two states. Light also initiates the generation of vitamin D, darkness generates melatonin—both are important hormones, neurotransmitters, and/or antioxidants that are essential for healthy sustainability of biologic systems [10-11].

Relationships determine the level of system functionality, which strongly depends on sensory processing and the eventual outcome of perception. One is automatic and subliminal, the other is volitional and fully reliant on cognition. Both processing and perception function interdependently within an endless cycle: processing of input, by sensory organs, in turn influences perception, which is created by the prefrontal cortex; its output has a forward influence on new sensory processing as it refocuses senses in a chosen direction. Activation of the senses generates awareness and presents the prefrontal cortex with choices of previously favored attractors (e.g. people, places, ideas, etc.) which perception now considers significant; some of these choices are motivated by health/sustainability, others by power, despair, etc. Once selected, the attractor becomes a dominant orientation of such a system and its method of interpretation of any new sensory input which may or may not be system optimizing. Knowledge creation is thus quite biased [12-13].

Decluttering, referring to a recycling process of outdated/non-contributing system components, must be active and ongoing and should encompass the “physical” as well as the “memory” accumulated items. This process of decluttering is critical for all cognition-determined relationships, because there is, indeed, an existing boundary threshold for speed and volume of sensory input within cognition of biologic systems. For humans, it runs only at about 60-100 bits per second, a paltry amount when compared to the massive capacity available to our senses. Anything above that number is lost to our perception, as it oversaturates cognition. Organizations are aggregates of individual biologic systems, so there also exists an organizational cognitive threshold, its combined capacity for input perception. Saturation of the threshold with white noise/irrelevant input, massively narrows the capacity for priority processing and perception, diminishing any ability to make optimized/rational decisions [14-15].

Not all systems are optimized and the differentiation among them comes from the character of their complexity which is determined by engaged relationships. How is the type of system’s complexity related to sustainability? Only organized complexity facilitates healthy sustainability. Any effort at enabling sustainability/persistence of non-optimized/unhealthy systems is detrimental to any related larger systems.

Nature in general and the human body in particular are strongly non-linear systems where fractal geometry is often visible to a naked eye. Why human thinking is still principally framed by the dominant concept of linearity, however, still awaits full explanation. And, here lies the dilemma: people with linear thinking, including many of its leaders, are attempting to live in and direct a world that is full of non-linear biologic systems; the resulting chronic dissonance is inescapable and the accompanying stress is an inevitable consequence.

A biologic system whose relationships and decisions are based on cognition have a significant advantage over other systems to achieve adaptation and evolution, but they can also evoke significant negative consequences for the self and future generations.

Our ability to decide, in spite of the presumed always-present “free will,” does vary greatly depending upon where any of us are within our personal life journey; it can be broadly divided into three time periods, termed the Phases of Life.

Phase 1: The Ancestral Phase runs from conception to young adulthood (about 25+ years). The dominant influence during this phase comes from: a) deterministic genes and the transgenerational epigenetically up/down regulated genes that come from parents and even older direct lineage based on their state of health; such an influence can manifest itself even through several future generations, b) microbiome received from mothers via vaginal delivery and/or breast feeding following birth; it creates the intestinal source of neurotransmitters that can be healthy or unhealthy, depending upon the composition of microbes, thus impacting the brain in likewise fashion [16].

In general, the features of time periods Phases 2 and 3 are mostly cultural expressions of specific cohorts and not a significant temporal determinism.

Phase 2: The Phase of Decisions spans the time frame from young adulthood to early seniority (about 50+ years). During this phase, the dominant influence on the self and others comes from: a) recent behavioral/lifestyle choices/decisions which may epigenetically either rebalance or worsen inherited set of gene functions or make new alterations; b) dietary decisions affecting the microbiome and generated neurotransmitters; c) the fitness level of the biologic system and its coping strategies for dealing with stress; d) when and where to share the existing features of epigenome and microbiomes with any new offspring via reproduction, thus expressing/continuing a transgenerational influence [17-18].

The impact of choices on the epigenome is well illustrated by the study of identical twins in Finland. They were initially raised together, but in adult life, during their Phase of Decisions, diverged in terms of the level of exercise.

This divergence was eventually reflected in different bodies and brains, in spite of identical genes. The sedentary twins had lower endurance capacities, higher body fat percentages, and signs of insulin resistance, signaling the onset of metabolic problems; the twins tended to have very similar diets. The active twins had significantly more grey matter than the sedentary twins, especially in areas of the brain involved in motor control and coordination. Overall, among healthy adult male twins in their mid-30s, a greater level of physical activity [was] associated with improved glucose homeostasis and modulation of striatum and prefrontal cortex gray matter volume, independent of genetic background [19].

Major genetic risk to a newborn, a new biologic system, likely reflects the transgenerational up/down epigenetic gene regulation created by the lifestyles of previous generations as well as those of the parental biologic systems.

Phase 3: The Phase of Consequences extends from early to late seniority (about 75+ years). The dominant influence here comes from the already “created” epigenome and microbiome during the previous phases. Better physical fitness in middle age, during the Phase of Decisions, for example, is reported to be associated with reduced cancer risk as well as mortality from cancer and cardiovascular disease after the age of 65 [20].

Chronologic time is not variable, as it is related to the unchanging number of the earth’s revolutions along its axis since a given birth. Contrary to that, biologic time is quite variable and currently can be estimated from the level of methylation of the epigenome as well as the length of the chromosomal telomeres; both existing cellular features are highly dependent on lifestyle choices, including diet, exercise, and stress management. Therefore, biologic age may be younger or older than chronologic age [21].

Systems science offers important advantages for assessing changes that we encounter daily as it is not a rule-based system, programmed by experts, who are likely strongly biased toward linear cause and effect thinking. On the contrary, system science embraces the probability of outcome in real time derived from the assessment of function and structure of any engaged system’s health. Systems science is free of bias and is fully transparent, contributing to its understandability.

The mechanics of our thinking vary greatly reflecting the variability and competence of our thought language, which may be primarily word-based, mathematical, statistical, code-based computational, musical, etc., depending upon how we construct our thoughts following the outcome of sensory perception. The electro-chemical signals of sensory processing that are taking place within the neuro-net are translated into sensory perception of our thoughts, our mind/consciousness.

“Free will” seemingly exists and makes the outcome of any human activity largely unpredictable thus representing a great challenge to artificial intelligence programs. The concept of free will rests on the assumption of being able to make unencumbered, willful choices. Those, however, are far from being free, as any choice is just an output of a biologic system, subject to all its limitations and proclivities. Systems science, however, offers forward-looking knowledge of the most likely ending probability of any encountered change. The evaluation process goes through decision tree questions: Did the encountered change originate from a healthy system generated by relationships of organized complexity? Is value creation likely through this change? Inputting such information into the Dynamic Systems Model makes the likely outcome quite visible.

Healthy systems are primarily oriented toward function, and structure then follows; unhealthy systems have proclivity for rigid structure with function as a distant second. DNA is biologic systems’ structure that is near-identical to all living entities. In nature, DNA is strongly deterministic on many levels and follows as heritable traits (e.g. in tomatoes, bananas, moss, etc. and even among primates where the determinism exists in organ formation, color of eyes, skin, etc.). In more complex cognitive systems, however, there exists greater functional influence on how the genetic structure behaves, offering a great versatility of function; this is accomplished via epigenetics and, as a consequence, humans need significantly smaller number of genes than other living entities, but they do need a great deal of optimizing decisions for healthy functioning. The epigenetic dynamics with various chemical tags create the up/down gene regulation, which is strongly influenced by lifestyle choices. As the complexity of systems increases, the breadth and depth of epigenetics increase as well. This process reaches a capstone in humans where the responsibility for the functional integrity of our structure, genes, is significantly delegated to the illusive ‘free will” in the Phases of Life.

The capacity for sustainability parallels the state of functional complexity of a studied system. Such a process, when healthy, engages in ongoing adaptation and evolution with life’s needs. For a human biologic system, it necessitates sensory processing, perception, and decision-making that are all congruent with such needs.

Healthy system-optimizing decisions must be based on commonly verifiable and generally accepted scientific facts, not opinions, in order to contribute to collective intelligence, rationality, and responsibility.

Biologic systems can only exist within ecologic climates that favors them; a non-optimized climate has a grossly negative influence on biologic systems and threatens their existence. Optimizing inter-related existence among climate and biologic systems is thus essential. A climate change induces adaptation and evolution of receptive biologic systems, which extracts its optimizing influence. Systems science reflects climate and its changes in the final larger-system probability assessment. Looking at climate change through a biologic system model, the human body, one can immediately see that focusing, for example, only on “rising temperature” is a single and possibly a misleading target as many subsequent decisions are slaves to chosen measures. The human body, the extrapolated system model, can present initially with rising temperature but that represents only a symptom, sometimes desirable and not a sign of a specific disease; an illness is much more complicated phenomena than a simple temperature chart and requires much broader conceptual understanding and corrective steps.

Health insurance for all is a very desirable societal goal. However, it is important to keep in mind that having health insurance does not equal good health. It seems somehow counterintuitive, at first, as all the insurance plans have the word “health” in their title or among their stated goals. It is a well-researched public health fact that a dominant group of illnesses is related to what is termed behavioral/lifestyle factors and only we, individually, have the real control over the myriad of decisions that lead to a potential salubrious outcome, not the insurance card.

The influence of industry on health care cost/outcomes/research, etc. is well known, documented, and usually reported as being negative. The pros and cons are sometimes hotly debated based on the writer’s orientation (philosophical, financial or both). For an optimized health care, each system component must actively contribute to the creation of the entire system’s emergence, its ultimate value. Yes, each component can make profit, etc. but its primary function cannot be, in a systems science term, self-maximizing.

Biologic systems as well as the entire ecology are as healthy as their components, from macro to micro scale. Each component part of living entities makes choices, either genetically-induced or cognition-determined; they may or may not be oriented toward the progress-regress rhythm of oscillating life cycles. Status quo, equilibrium, does not exist among open systems.

Life has an inherent bias toward sustainability; our “free will” should move in the same direction.

Systems science makes visible a matrix for a healthy system, small or large. Only healthy systems are sustainable, able to adapt and evolve harmoniously with change. To function outside of systems science, the framework for life encounters increased randomness, ever-mounting non-functionality, inability to adapt and evolve with change, and a failing aptitude for sustainable predictions.

Sustainability of healthy biologic systems demonstrates correlation with life cycles.

Three-dimensional nature-available sensory input of a biologic system is highly preferable to an artificial 2-D, flat screen one, which leads to poor patterns of memory formation, deprived probability comparison, and reduced cognitive outcome/ decisions.

Memory is the beginning and the end of a cognitive life. Executive prefrontal cortex is capable of creating an internal dialog, the thought language of the mind, all based on past memory and a new sensory input.

The Dynamic Systems Model allows for the categorization of systems into healthy and unhealthy ones. It is accomplished through the examination of pertinent/engaged systems’ function and structure. The spectrum of differentiating systems’ features within the above model include the following.

*Healthy systems *express function which precedes structure. Organized complexity, manifesting self-organization, represents learning relationships and active feed-back loops. Healthy attractors are selected from sensory awareness and no error lasts too long unchecked. The majority of uncertainty is absorbed and converted into a quantifiable risk. Predictions are highly accurate and neuroplasticity is high. Component ratios are homeostatic/system-optimizing and value is created; overall, there is a system coherence. Sensory processing develops 3-D memory patterns in the hippocampus and continues to the prefrontal cortex for preferential system-optimizing decisions.

*Unhealthy systems, those in Chaos, *express function that follows structure. There is disorganized complexity with exponential growth, lack of differentiation, and irrational exuberance. There are failing relationships that select attractors of power; prediction error is large and errors last too long. Neuroplasticity is negatively altered; uncertainty prevails, creating component ratios that are not homeostatic/system-optimizing and value is not created. Overall, there is system incoherence. Sensory processing does not develop 3-D memory patterns in the hippocampus and continues, preferentially, to the prefrontal cortex via reward pathway for unlikely system-optimizing decisions.

*Unhealthy systems, those in Entropy, *express a state where both function and structure are regressing. There is diminishing complexity and vanishing growth. Non-functionality rises with diminished decluttering/recycling resulting in waning relationships; attractors of despair are selected/preferred and there is an extended apathy. Neuroplasticity is gone; predictions are seldom made and are not accurate. Value is not created and the entire system is closing. Sensory processing does not develop 3-D memory patterns in the failing hippocampus and continues to the weakened prefrontal cortex via reward pathway for unlikely system-optimizing decisions.

### Health and systems science: a framework

1st- Health is not health care. Health care may or may not help to reestablish health but the terms are far from being synonymous.

2nd- Health is a system’s emergence/outcome, either positive, good health or negative, poor health.

3rd- Emergence is a transient state requiring continuous adaptation and evolution.

4th- Healthy emergence requires that its system generates organized complexity through healthy relationships on all levels.

5th- Organized complexity reflects the participation of all of its diverse components that are engaged in feedback and communication, expressed in self-organization, as an outcome of system-optimizing steps.

6th- Health is contextual, expressing systems’ inter-relatedness, which is a characteristic of open systems with input-throughput-output of gradient flow of energy and information. One system’s input is another system’s output.

7th- No amount of healthcare can guarantee lasting health, reflecting the old adage: doctors treat but patients do the healing.

8th- Health is related to genetics, socio-economic status, education, diet, lifestyle, etc. But, it’s not the “genes” or the “veggies” or even the “insurance card” that start or stop the cascade toward health or illness; it’s our decisions.

9th- Decisions are based on our cognition, an expression of level of collaboration between the memory and the executive cortex, guiding all epigenetic changes.

10th- Simply, your body speaks your hippocampus and the prefrontal cortex.

There is an immediate and implementable solution to the escalating cost of health care. Combine health care premium with a life insurance payable to whoever is the health insurance underwriter; tables for life expectancy, risk stratification, etc. are already available and historically tested. The phase-in period could be subsidized, but in the not too distant future, the health care cost would morph into a zero-sum game as the cost for unhealthy/sick people, who live fewer years, is the same as the cost for healthy people who live longer.

Health care is a system in its own right and thus can be either “healthy” or “unhealthy”, optimized or non-optimized, as judged by its compliance with systems science principles.

Our individual as well as collective health is dependent on dynamic, collaborative, system-optimizing functional relationships of our sub-systems/components engaged in self-organization and adaptation to the external and the internal environment. Health is a personal matter with societal consequences; health care is societal endeavor with personal impact.

An open biologic system is subjected to many variables affecting its status. An optimal state, referred to as its health, is a non-linear expression of myriad of decisions, ours as well as those that preceded us and who left their imprint on the ever-changing epigenome. A bodily phenotype is its morphologic expression.

Health-related statistics for the entire societal system indicate significant dysfunction on a large scale, showing great dominance of chaos and entropy-related illnesses, exemplified by cancer in chaos and degeneration in entropy. True health and fitness are rare.

An ongoing process of system decluttering/recycling is essential for the continuity of health of any biologic system. Without it, the process of aging, seen as accumulation of non-functionality, an Entropic state within the Dynamic Systems Model, prevails as the dominant state/expression of a biologic system. Epigenetic changes create system clutter and it is likely that only new pro-health epigenetic changes can guide the decluttering/recycling process. Among cognition-based biologic systems, decisions create the majority of determining/influential lifestyle choices.

There is one preventive step that is so cost effective and so efficacious that a society should always measure any interventions against it. It is the value of physical activity. For example, physical activity and colon cancer are engaged in a reciprocal relationship. As one goes down, the other rises and vice versa. The reason is that physical activity exerts its positive influence in all of the currently known colon cancer-causing categories and without side effects. It is unlikely that a pill can ever duplicate that.

The best example of a complex adaptive system is the human body which exemplifies systems science principles in health. It is possible to compare complex adaptive system, current health care, and goals for solutions. Each column listed in Table 1 indicates what is present in a complex adaptive system, what is absent in the current health care, and where corrective efforts should be focused, listed as solutions (Table 1).

**Table 1:** Comparison of Characteristics Found in Systems Science, Health Care, and Needed Solutions.

COMPLEX SYSTEM	HEALTH CARE	SOLUTIONS
Numerous & variable components	Not all components are included	All must be included
Generates emergence/value	Value is inconsistent	Value to be created
All components are responsible	Diffuse responsibility	Responsibility: individual for health, societal for healthcare
Relationships optimized with feedback loops	Relationships are not pro-system	Integrate all components
Organized complexity characterizes function	Organization is failing	Organized complexity
Communication uses uniform language and resembles a self-learning neuro-net	Choices are not based on existing knowledge	Uniformity of communication, life-long education
Open system seeks homeostasis/steady state	Choices override needs	Set balance via diet, exercise, stress management
Balance of function leads to cost/energy stability	Health/disease imbalance increases cost	Combine life and health insurance
Horizontal and vertical hierarchies are reciprocal	Vertical hierarchy controls	Reestablish reciprocity; create system-optimizing policies/tort
Boundary is semipermeable, in sync with external environment	Poor adaptation to environment	Flexible adaptation
All systems inter-relatedness exists	Variable systems don't relate	Connect all systems
Systems' cycles are intelligently reset	Resetting through crises	Introduce rational resetting
Systems are of proportionate size to maintain viability of feedback loops, semipermeability of boundary, and actionable resetting	Disproportionality exists	Allow re-sizing of healthcare units/subsystems
Systems show growth balance/ outcome of reciprocal hierarchies	Imbalance in growth	Formulate mission/goals/ authority/functional space for each hierarchy

## Conclusions

Healthy sustainability is related to the way systems deal with change, individually and collectively; when present, health is the output, the emergence, of an optimized system. In order for a biologic system to create healthy emergence, it first needs to select optimizing attractors for its sensory processing that exists in the field of overall awareness. Second, the sensory perception that follows needs to engage the collaboration of 3-D hippocampal memory and an optimized executive prefrontal cortex. An alternate, though unhealthy, pathway does exist, when sensory input is processed through the reward center of the neuro-net instead of the hippocampus and ends in the prefrontal cortex that is in a state of a dysexecutive syndrome.

This research supports the following concepts expressed as hypotheses:

For cognition-dependent systems, not everything that seems logical to sustain for health should be so. Only healthy/optimized biologic systems can sustain themselves.

Systems science is highly capable of differentiating optimized from non-optimized systems, those that create healthy sustainability from those that do not.

The human body has been identified as the best complex-adaptive system and can serve as a model for judging other biologic systems.

The hippocampal memory and the executive prefrontal cortex loop is instrumental in making healthy decisions.

A healthy society can only emerge when the societal system components—individuals, corporations, government—are healthy, system optimizing and not self-maximizing, with a horizontal hierarchy—the “people in the trenches” being allowed to “self-organize”—and be supported by the “vertical/governing hierarchy” that is passing and enforcing laws that optimize the societal system, thus allowing health, as a system outcome, to emerge.

Actionable guidelines: become an optimized system, one that is fit and creates value. As a consequence, the large societal system will emerge healthy when the prevailing number of components achieve their own health status.

## Appendices

For further perspectives on biologic systems and systems science, please see:

Janecka IP: Is the U.S. health care an appropriate system? A strategic perspective from systems science. Health Research Policy and Systems 2009, 7:1 (Highly Accessed) http://www.health-policy-systems.com/content/7/1/1

Janecka IP: Cancer control through principles of systems science, complexity, and chaos theory: a model. Int J Med Sci 2007; 4:164-173. http://www.medsci.org/v04p0164.htm

Janecka IP: Prevention and Management of Colon Cancer through Physical Activity. VDM Verlag, Saarbrücken, Germany. ISBN 978-3-639-01696-3
